# Paving the way for the oncological process in patients with schizophrenia

**DOI:** 10.1192/j.eurpsy.2021.1978

**Published:** 2021-08-13

**Authors:** F. Casanovas, J.I. Castro, C. Masferrer, L. Martínez, V. Gallardo, M.D.L.Á. Sotomayor, F. Dinamarca, O. Orejas, S. Oller, V. Pérez-Solà

**Affiliations:** Institut De Neuropsiquiatria I Addiccions (inad), Hospital del Mar, Barcelona, Spain

**Keywords:** peer-support, schizophrénia, oncology

## Abstract

**Introduction:**

Oncologic patients with schizophrenia have a higher mortality, which could be explained by a delayed diagnosis and a poor quality of the oncologic treatment (1). Some of the potential reasons are related with patient’s psychopathology, stigma, and barriers in access to medical care. An structured support during the oncologic treatment has been proposed to solve the difficulties that patients with schizophrenia can experience when handling with an oncologic process. (2).

**Objectives:**

To illustrate two approaches for cancer accompaniment in patients with schizophrenia.

**Methods:**

We present two case-report and literature research of the topic.

**Results:**

Case A. A 49 y.o. woman diagnosed with a schizoaffective disorder. In the last years she had difficulties to manage her selfcare, so her mental health providers linked her to an individualized community nurse, who later played a crucial role in helping the patient during the diagnosis and treatment of a breast cancer. Case B. A 37 y.o. man diagnosed with schizophrenia, who was very integrated in a peer-support organization. After being diagnosed with a Lymphoma, he continued participating in all the group activities (theatre, collaborative radio, painting) until his decease. Sharing the process with other patients not only improved his quality of life but also helped the group to manage the grief.

**Conclusions:**

- Individualized support with a mental health nurse could enhance the communication between the oncologist and mental health providers, improve the symptoms management, and allow psychological support. - Peer-support can prevent social isolation, improve the quality of life and the management of the oncologic treatment.

**Disclosure:**

No significant relationships.

